# Case Report: A Novel Heterozygous Mutation of *CD2AP* in a Chinese Family With Proteinuria Leads to Focal Segmental Glomerulosclerosis

**DOI:** 10.3389/fped.2021.687455

**Published:** 2021-08-02

**Authors:** Yu-Xing Liu, Ai-Qian Zhang, Fang-Mei Luo, Yue Sheng, Chen-Yu Wang, Yi Dong, Liangliang Fan, Lv Liu

**Affiliations:** ^1^Department of Obstetrics and Gynecology, The Third Xiangya Hospital of Central South University, Changsha, China; ^2^Department of Cell Biology, The School of Life Sciences, Central South University, Changsha, China; ^3^Hunan Key Laboratory of Animal for Human Disease, School of Life Sciences, Central South University, Changsha, China; ^4^Department of Respiratory Medicine, Diagnosis and Treatment Center of Respiratory Disease, The Second Xiangya Hospital of Central South University, Changsha, China

**Keywords:** FSGS, CD2AP, mutation, heterozygote, whole-exome sequencing

## Abstract

Idiopathic focal segmental glomerulosclerosis (FSGS) is a relatively frequent kidney disorder that manifest clinically as proteinuria and progressive loss of renal function. Genetic factors play a dominant role in the occurrence of FSGS. CD2-associated protein (CD2AP) is an adapter molecule and is essential for the slit-diaphragm assembly and function. Mutations in the *CD2AP* gene can contribute to FSGS development. Here, we describe a Chinese family of four generations with unexplained proteinuria. The proband, a 12-year-old boy, was diagnosed as FSGS. Whole-exome sequencing (WES) revealed an unknown frameshift insertion mutation (p.K579Efs^*^7) of *CD2AP* gene that leads to a truncation of CD2AP protein. Bioinformatics strategies predicted that the novel mutation was pathogenic. The mutation was absent in either healthy family members or our 200 healthy controls. In summary, we used WES to explore the genetic lesion of FSGS patients and identified a novel mutation in *CD2AP* gene. This work broadens the mutation spectrum of *CD2AP* gene and provides data for genetic counseling to additional FSGS patients.

## Introduction

Idiopathic focal segmental glomerulosclerosis (FSGS) is a relatively frequent kidney disorder that manifests clinically as proteinuria and progressive deterioration of renal function. FSGS is histologically characterized by focal and segmental glomerular sclerosis and foot-process effacement (1). As a leading cause of steroid-resistant nephrotic syndrome (SRNS), FSGS makes up about three quarters of the SRNS in children and adults and frequently leads to end-stage renal disease (2).

Genetic factors play a dominant role in the occurrence and development of FSGS. As a kind of podocytopathy, many FSGS-causing genes have been identified and are mainly expressed in glomerular podocytes. The proteins encoded by these genes are crucial for the maintenance of podocyte structure and function, including protein assembly of glomerular basement membrane (GBM) and podocyte skeleton (1). Over the past decades, at least 60 genes have been linked to SRNS (3). Among them, ~20 pathogenic genes have been identified in FSGS patients (4). Genes such as collagen α3-5 (*COL4A3–5*), anillin actin binding protein (*ANLN*), inverted formin 2 (*INF2*), paired box 2 (*PAX2*), transient receptor potential cation channel 6 (*TRPC6*), α-actinin-4 (*ACTN4*), and podocin (*NPHS2*) show a higher mutation rate in FSGS patients (5–10). Moreover, mutations of CD2 associated protein (*CD2AP*) and Rho GTPase activating protein 24 (*ARHGAP24*) can also contribute to FSGS development (11, 12). With the development of sequencing technology, more causative genes, such as IFT139 (*TTC21B*), LIM homeobox transcription factor 1β (*LMX1B*), Integrin subunit β4 (*ITGB4*), and Nuclear RNA export factor 5 (*NXF5*), were found in rare FSGS (13–15). On account of high genetic heterogeneity and complicated hereditary constitution, the genetic etiology of primary FSGS remains obscure in many cases.

Herein, we investigate a Chinese family with unexplained proteinuria. The proband was diagnosed as FSGS. Using whole-exome sequencing (WES) technology in combined with bioinformatics strategies, we detected a previously unreported heterozygous mutation in *CD2AP*.

## Case Presentation

### Clinical Features

**Figure 1:**
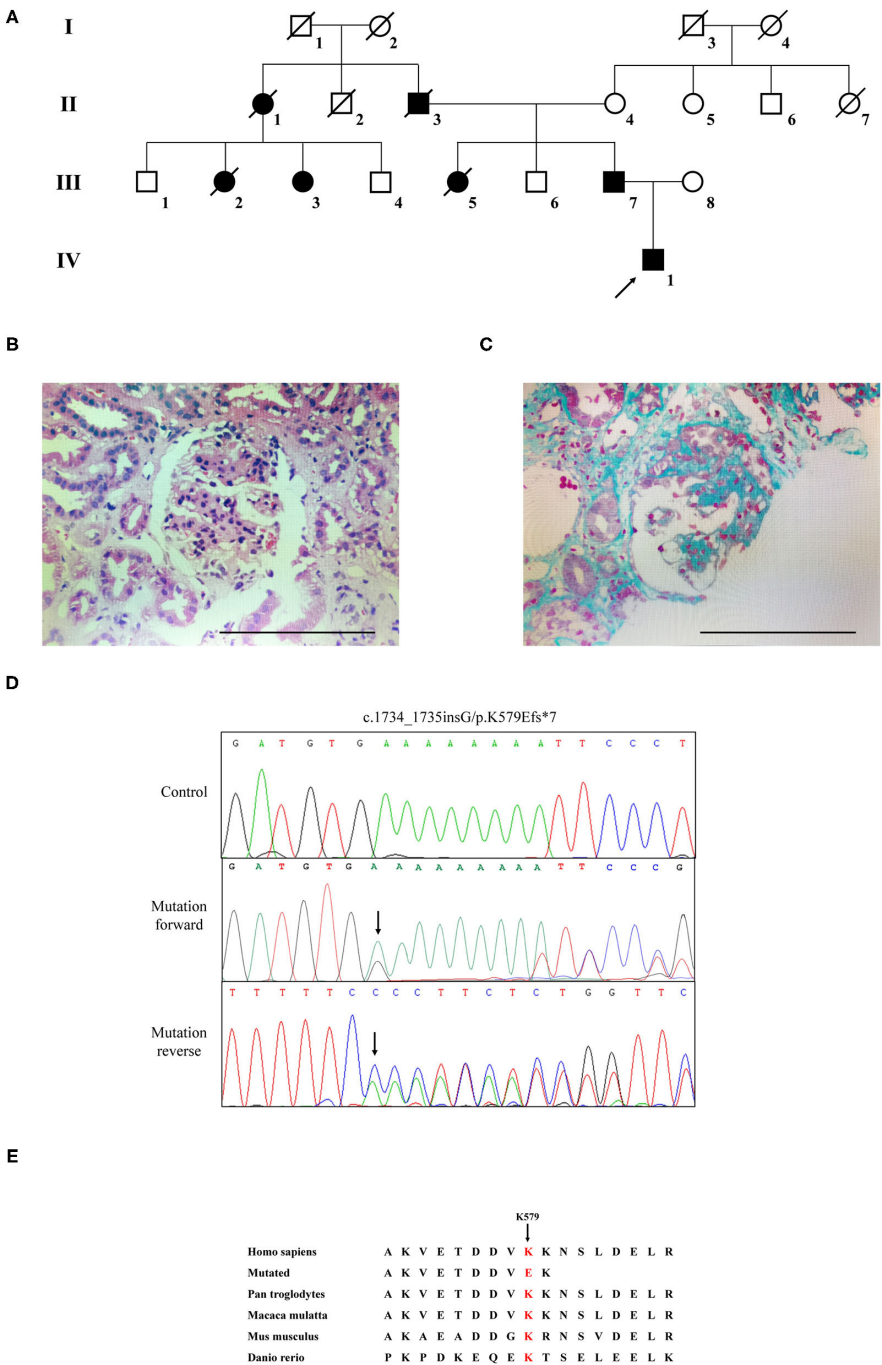
The clinic and sequencing data of this FSGS family. **(A)** The pedigree of this FSGS family. Squares indicate male family members; circles, female members; black symbols, the affected members; white symbols, unaffected members; arrow, proband. **(B)** HE staining **(C)** Masson staining for renal biopsy of the proband (IV-1). **(D)** Sanger DNA sequencing chromatogram demonstrates a heterozygosity mutation (c.1734_1735insG/p.K579Efs*7) of *CD2AP* gene in affected members. **(E)** Alignment of multiple CD2AP protein sequences across species. Letters in red show the K579 site is evolutionarily conserved.

The Chinese family with four generations including 13 persons was described in this research (Figure 1A). Three living cases (IV1, III-3, and III-7) among the seven patients were enrolled in this family. Two hundred unrelated healthy subjects were collected as control subjects to exclude polymorphisms. The information of the healthy controls group has been provided in our previous study (16).

The proband (IV1) was a 12-year-old boy from Hunan Province in China. He visited our hospital because of an abnormal urine test. Physical examination showed lower extremity edema and hypertension. Laboratory examination showed high proteinuria (2+), high serum creatinine (149 μmol/L), blood urea nitrogen (8.81 mmol/L), and uric acid (478.6 μmol/L). Microscopic urine analysis indicated microscopic hematuria (1+). Since persistent proteinuria after 6 weeks of prednisone treatment (60 mg/m^2^/day), the patient was considered as steroid resistant. Renal biopsy was carried out and revealed glomerulomegaly, segmental podocytes proliferation, and hypertrophic. The GBM characterized segmental thickening. Masson staining mesangial area showed mesophilic deposition (Figures 1B,C). Thus, the patient was diagnosed as FSGS. Family history survey showed his father (III-7) also suffered from proteinuria. One aunt (III-3) investigated proteinuria and had a similar 3-year history of lower extremity edema and high blood pressure. The proband's mother refused further medical examination because of divorce. The relevant clinical data of the patients in this family are provided in Table 1.

**Table 1:** Clinical data of three patients in this family.

**Subjects**	**IV-1 (proband)**	**III-3**	**III-7**	**Normal**
Sex	M	F	M	/
Age (years)	12	49	40	/
Microscopic hematuria	1+	-	-	-
Proteinuria	2+	1+	1+	-
Uraemia	No	No	No	/
Blood creatinine (μmol/L)	149.0	128.3	168.5	M: <106; F: <86
Blood urea nitrogen (mmol/L)	8.81	11.04	8.52	1.8-7.1
Uric acid (μmol/L)	478.6	520.5	444.3	M:149-416; F: <89-357

*F, female; M, male*.

### Genetic Analysis

**Figure 2:**
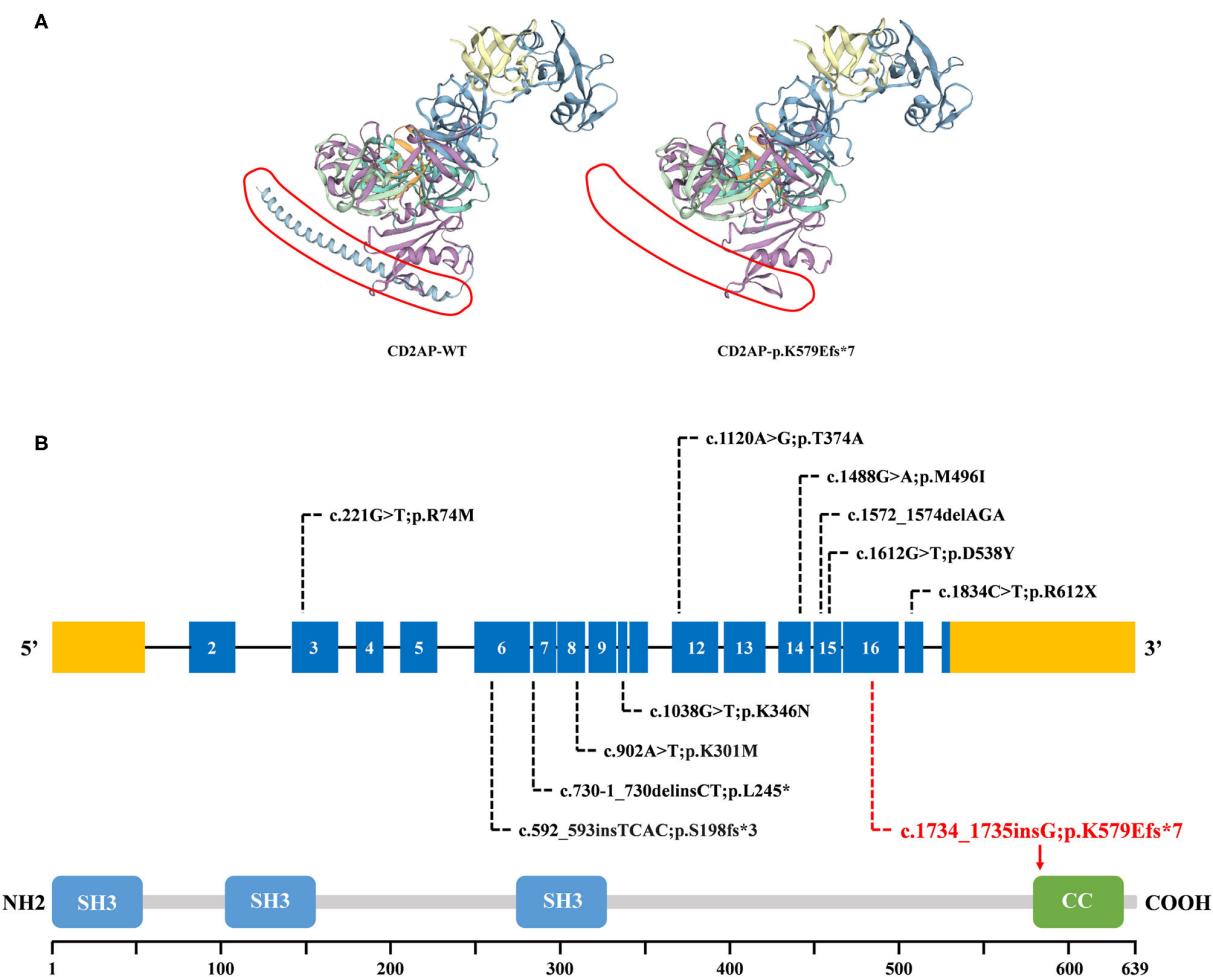
The bioinformatics analysis of mutations. **(A)** Structure prediction of the mutant protein. The wild type CD2AP (CD2AP-WT) protein structure and the p.K579Efs*7 mutant CD2AP (CD2AP-p.K579Efs*7) protein structure were predicted by SWISS-MODEL online software. **(B)** Overview of all known and novel CD2AP mutations. The *CD2AP* gene is shown, with all known *CD2AP* mutations (black letters) and novel mutation (red letters). Blue rectangles indicate exons. Introns are not shown to scale. The CD2AP protein structure is shown. CC, coiled-coil domain; SH3, Src homology 3 domain.

WES yielded 9.13 Gb of data with 99.7% coverage of the target region and 99.1% of the target covered over 10×. In total, about 4,995 variants were detected in the proband. Data filtering were performed as our previous study (17). A set of 11 variants in 11 genes were detected (Table 2) and were further analyzed. Information including the inheritance pattern, OMIM clinical phenotypes, Toppgene function (18), and American College of Medical Genetics Classification (19) of these 11 genes has been shown in Table 2. No variants in other known FSGS-related genes were detected. Sanger sequencing was carried out in all family members. Co-segregation analysis shown that only a previously not described heterozygous mutation (c.1734_1735insG/p.K579Efs^*^7) in exon 16 of the *CD2AP* gene was observed in all three affected patients (III-3, III7, and IV-1) and excluded in the healthy members (Figure 1D). Family screening showed that the frameshift mutation was inherited *via* the paternal allele (Figure 1A). The newly identified mutation was absent in our 200 healthy controls. Alignment of CD2AP amino acid sequences revealed the affected amino acid was evolutionarily conserved (Figure 1E). In addition, Swiss-Model software (https://swissmodel.expasy.org/interactive) was utilized to explore the spatial configuration of this CD2AP mutation. As the results showed, a loss of almost all of the C-terminal in the K579Efs^*^7 mutated CD2AP protein was observed, in comparison with the wild type, as marked by the red frame in the figure (Figure 2A).

**Table 2:** Variants identified by WES in this family.

**Gene**	**Transcript variant**	**Protein variant**	**SIFT**	**Polyphen-2**	**Mutationtaster**	**GnomAD**	**OMIM clinical phenotype**	**ToppGene function**	**American college of medical genetics classification**
*SLC28A1*	NM_004213.4; c.1-16C>G	-	-	-	D	0.0189213	AR, uridine-cytidineuria	Purine nucleobase transport	pvs1, pm3, pp3
*NEUROD1*	NM_002500.4; c.34G>C	p.G12R	T	B	D	0.0015766	AD, type 2 diabetes mellitus	Pancreatic A cell fate commitment	ps1, pm2
*TGM6*	NM_198994.2; c.1171G>A	p.V391M	D	B	D	0.0096227	AD, spinocerebellar ataxia	Peptide cross-linking	ps1, pm1, pp3
*CIDEC*	NM_022094.3; c.457C>T	p.Q153*	-	-	D	-	AR, lipodystrophy	Lipid droplet organization	pvs1, pm1, pm2
*TREM2*	NM_018965.3; c.574G>A	p.A192T	T	B	P	0.000435114	AR, Polycystic lipomembranous osteodysplasia with sclerosing leukoencephalopathy	C-C chemokine receptor CCR7 signaling pathway	ps1, pm2, bp4
*HOXA10*	NM_018951.3; c.170A>G	p.Y57C	T	D	D	0.00121275	-	Proximal/distal pattern formation	ps1, pm2
*CFTR*	NM_000492.3; c.1666A>G	p.I556V	T	B	D	0.0471606	AR, Cystic fibrosis	Regulation of cyclic nucleotide-gated ion channel activity	ps1, pm1
*PYGM*	NM_005609.3; c.1860del	p.I621Sfs*37	-	-	D	-	AR, McArdle disease	Glucan catabolic process	pvs1, pm2
*MCTP2*	NM_018349.3; c.239del	p.S80Tfs*17	-	-	D	0.00364805	-	Calcium-dependent phospholipid binding	pvs1, pm2
*XIRP2*	NM_152381.5; c.3318_3319del	p.Y1107*	-	-	D	-	-	Muscle tissue morphogenesis	pvs1, pm2
*CD2AP*	NM_012120.2; c.1734dup	p.K579Efs*7	-	-	D	-	AD, Glomerulosclerosis, focal segmental	Transforming growth factor beta1 production	pvs1, pm2

*B, begin; D, disease-causing; P, polymorphism; T, tolerated; AD, autosomal dominant; AR, autosomal recessive; bp, pathogenic benign; pp, pathogenic supporting; ps, pathogenic strong; pvs, pathogenic very strong; pm, pathogenic moderate*.

## Discussion

In the current research, we described a Chinese family with unexplained proteinuria. The proband was diagnosed as FSGS. Employing WES combined with bioinformatics strategies, a newly heterozygous mutation (p.K579Efs^*^7) of the *CD2AP* gene was detected. The frameshift mutation (p.K579Efs^*^7) locates in the exon 16 that alters the lysine codon at position 579 to a glutamic codon, and is expected to form a premature stop codon, leading to a truncated protein. Sanger sequencing confirmed that all 3 affected members in this FSGS family, including the proband's father (III-7) and his aunt (III-3), harbored the heterozygous frameshift mutation in *CD2AP*. The late patients' DNA was not available. Moreover, this heterozygous variant did not exist in the remaining unaffected family members. Given the segregation of the frameshift mutation with the disease phenotype and the degree of protein structure alteration, it was highly considered that the mutation was responsible for the FSGS in this family.

FSGS is clinically characterized by proteinuria and progressive renal failure (1). In our study, all living patients (III-3, III7, and IV-1) in this family presented proteinuria, but definitive data on progression to end-stage renal failure were not available. In a previous report, Gigante et al. screened for changes in the *CD2AP* gene in a total of 80 Italian patients with idiopathic nephrotic syndrome. Three heterozygous mutations in *CD2AP* gene were found in three unrelated patients while there were no definitive data on renal failure in the reported patients (20). For the different molecular and pathogenesis bases of genetically associated FSGS and SRNS, the manifestation and prognosis are different (1). Thus, the genotype-phenotype relation between *CD2AP* gene and FSGS needs to be further investigated. Since most FSGS/SRNS patients with genetic factors do not respond to common treatment and show poor prognosis (1), the patients in this family were given the cyclosporine treatment according to the KDIGO 2012 guideline (21). And a follow-up visit has been scheduled in a few months to ensure the patients benefit from treatment.

CD2AP is prominently expressed in glomerular podocytes. It is an 80 kDa cytoplasmic protein which consists of four domains: three Src homology 3 (SH3) domains at the NH2 terminus and one coiled-coil domain at the COOH terminus (22). CD2AP was initially identified as a ligand for the T-cell-adhesion protein CD2. And it was also shown to bind to nephrin and podocin, thereby acting as an important component of the slit-diaphragm (SD) network (23). The three SH3 domains are essential for the interaction of CD2AP protein with CD2 (20). Recent reports exploring that phosphorylation of tyrosine residues within the SH3–1 domain may modify interactions between CD2AP and its binding partners, including nephrin (24). Furthermore, it has been demonstrated that CD2AP directly interacts with nephrin at the C-terminal region between amino acids 428 and 600 (25, 26). As shown in Figures 2A,B, compared with 639 amino acids in the wild type, the resulting truncated protein is 580 amino acids in length with an abnormal binding domain of nephrin, and lacks the coiled-coil domain that promotes homodimerization. In this case, the truncated CD2AP protein in our study would fail to bind to nephrin. Similarly, Gigante et al. reported a frameshift mutation (p.delE525) in *CD2AP*, which is localized in the same region, affects the ultrafiltration functions of the SD network and might lead to proteinuria (20). Thus, we indicate that the frameshift mutation (p.K579Efs^*^7) identified in *CD2AP* gene may be a potential candidate factor for the development of FSGS, consistent with the previous research.

*CD2AP* was a strong candidate gene for nephrotic syndrome (NS). Animal studies have shown that *CD2AP* knockout mice suffer from severe NS and die of massive proteinuria in infancy (27). Moreover, the *CD2AP* heterozygous mouse is prone to proteinuria and presents a glomerular disease at old age with a histology pattern that is similar to human FSGS (28). The relevance of *CD2AP* in human renal pathology still remains largely unknown and the detailed molecule mechanisms involved requires further investigation (20). So far, ~10 mutations of *CD2AP* have been reported in FSGS or NS patients. A brief review of these reported mutations was shown in Figure 2B, which may help for the genetic counseling and prenatal diagnosis of FSGS associated with mutation in the *CD2AP* gene. Although the pathogenic mechanism involved still requires further investigation, our findings offer more evidence that *CD2AP* gene variant is significant in FSGS. Remarkably, the mutation (c.1734_1735insG/p.K579Efs^*^7) identified in this study has not been published and, therefore, is considered novel.

## Conclusion

We applied WES to explore the genetic lesion in a Chinese FSGS family. A novel heterozygous mutation (c.1734_1735insG/p.K579Efs^*^7) of *CD2AP* was identified. Our study broadens the mutation spectrum of *CD2AP* gene and provides data for the clinical management and genetic counseling respect to FSGS.

## Data Availability Statement

The datasets presented in this study can be found in online repositories. The names of the repository/repositories and accession number(s) can be found below: NCBI BioSample; PRJNA739264.

## Ethics Statement

The studies involving human participants were reviewed and approved by the Third Xiangya Hospital of Central South University. Written informed consent to participate in this study was provided by the participants' legal guardian/next of kin. Written informed consent was obtained from the individual(s), and minor(s)' legal guardian/next of kin, for the publication of any potentially identifiable images or data included in this article.

## Author Contributions

Y-XL and A-QZ enrolled the family members. F-ML and YS performed DNA isolation and sanger sequencing. C-YW and YD performed genetic analysis. Y-XL and LF wrote the manuscript. LL supported the project. All authors reviewed the manuscript.

## Conflict of Interest

The authors declare that the research was conducted in the absence of any commercial or financial relationships that could be construed as a potential conflict of interest.

## Publisher's Note

All claims expressed in this article are solely those of the authors and do not necessarily represent those of their affiliated organizations, or those of the publisher, the editors and the reviewers. Any product that may be evaluated in this article, or claim that may be made by its manufacturer, is not guaranteed or endorsed by the publisher.

## References

[1] Liu J Wang W. Genetic basis of adult-onset nephrotic syndrome and focal segmental glomerulosclerosis. Front Med. (2017) 11:333–9. 10.1007/s11684-017-0564-1 28776307

[2] Lowik M Levtchenko E Westra D Groenen P Steenbergen E Weening J. Bigenic heterozygosity and the development of steroid-resistant focal segmental glomerulosclerosis. Nephrol Dial Transplant. (2008) 23:3146–51. 10.1093/ndt/gfn208 18443213

[3] Saleem MA. Molecular stratification of idiopathic nephrotic syndrome. Nat Rev Nephrol. (2019) 15:750–65. 10.1038/s41581-019-0217-5 31654044

[4] Fan LL Liu L Luo FM Du R Wang CY Dong Y. A novel heterozygous variant of the COL4A4 gene in a Chinese family with hematuria and proteinuria leads to focal segmental glomerulosclerosis and chronic kidney disease. Mol Genet Genomic Med. (2020) 8:e1545. 10.1002/mgg3.1545 33159707 PMC7767549

[5] Gast C Pengelly RJ Lyon M Bunyan DJ Seaby EG Graham N. Collagen (COL4A) mutations are the most frequent mutations underlying adult focal segmental glomerulosclerosis. Nephrol Dial Transplant. (2016) 31:961–70. 10.1093/ndt/gfv325 26346198

[6] Xie J Hao X Azeloglu EU Ren H Wang Z Ma J. Novel mutations in the inverted formin 2 gene of Chinese families contribute to focal segmental glomerulosclerosis. Kidney Int. (2015) 88:593–604. 10.1038/ki.2015.106 26039629

[7] Barua M Stellacci E Stella L Weins A Genovese G Muto V. Mutations in PAX2 associate with adult-onset FSGS. J Am Soc Nephrol. (2014) 25:1942–53. 10.1681/ASN.2013070686 24676634 PMC4147972

[8] Zhang Q Ma J Xie J Wang Z Zhu B Hao X. Screening of ACTN4 and TRPC6 mutations in a Chinese cohort of patients with adult-onset familial focal segmental glomerulosclerosis. Contrib Nephrol. (2013) 181:91–100. 10.1159/000348471 23689571

[9] Gbadegesin RA Hall G Adeyemo A Hanke N Tossidou I Burchette J. Mutations in the gene that encodes the F-actin binding protein anillin cause FSGS. J Am Soc Nephrol. (2014) 25:1991–2002. 10.1681/ASN.2013090976 24676636 PMC4147982

[10] He N Zahirieh A Mei Y Lee B Senthilnathan S Wong B. Recessive NPHS2 (Podocin) mutations are rare in adult-onset idiopathic focal segmental glomerulosclerosis. Clin J Am Soc Nephrol. (2007) 2:31–7. 10.2215/CJN.02690806 17699384

[11] Lowik MM Groenen PJ Pronk I Lilien MR Goldschmeding R Dijkman HB. Focal segmental glomerulosclerosis in a patient homozygous for a CD2AP mutation. Kidney Int. (2007) 72:1198–203. 10.1038/sj.ki.5002469 17713465

[12] Akilesh S Suleiman H Yu H Stander MC Lavin P Gbadegesin R. Arhgap24 inactivates Rac1 in mouse podocytes, and a mutant form is associated with familial focal segmental glomerulosclerosis. J Clin Invest. (2011) 121:4127–37. 10.1172/JCI46458 21911940 PMC3195463

[13] Esposito T Lea RA Maher BH Moses D Cox HC Magliocca S. Unique X-linked familial FSGS with co-segregating heart block disorder is associated with a mutation in the NXF5 gene. Hum Mol Genet. (2013) 22:3654–66. 10.1093/hmg/ddt215 23686279

[14] Huynh CE Bizet AA Boyer O Woerner S Gribouval O Filhol E. A homozygous missense mutation in the ciliary gene TTC21B causes familial FSGS. J Am Soc Nephrol. (2014) 25:2435–43. 10.1681/ASN.2013101126 24876116 PMC4214529

[15] Boyer O Woerner S Yang F Oakeley EJ Linghu B Gribouval O. LMX1B mutations cause hereditary FSGS without extrarenal involvement. J Am Soc Nephrol. (2013) 24:1216–22. 10.1681/ASN.2013020171 23687361 PMC3736714

[16] Xiang R Fan LL Huang H Cao BB Li XP Peng DQ. A novel mutation of GATA4 (K319E) is responsible for familial atrial septal defect and pulmonary valve stenosis. Gene. (2014) 534:320–3. 10.1016/j.gene.2013.10.028 24498650

[17] Fan LL Liu JS Huang H Du R Xiang R. Whole exome sequencing identified a novel mutation (p.Ala1884Pro) of beta-spectrin in a Chinese family with hereditary spherocytosis. J Gene Med. (2019) 21:e3073. 10.1002/jgm.3073 30690801

[18] Kaimal V Bardes EE Tabar SC Jegga AG Aronow BJ. ToppCluster: a multiple gene list feature analyzer for comparative enrichment clustering and network-based dissection of biological systems. Nucleic Acids Res. (2010) 38:W96–102. 10.1093/nar/gkq418 20484371 PMC2896202

[19] Richards S Aziz N Bale S Bick D Das S Gastier-Foster J. Standards and guidelines for the interpretation of sequence variants: a joint consensus recommendation of the American College of Medical Genetics and Genomics and the Association for Molecular Pathology. Genet Med. (2015) 17:405–24. 10.1038/gim.2015.30 25741868 PMC4544753

[20] Gigante M Pontrelli P Montemurno E Roca L Aucella F Penza R. CD2AP mutations are associated with sporadic nephrotic syndrome and focal segmental glomerulosclerosis (FSGS). Nephrol Dial Transplant. (2009) 24:1858–64. 10.1093/ndt/gfn712 19131354

[21] Chen YM Liapis H. Focal segmental glomerulosclerosis: molecular genetics and targeted therapies. BMC Nephrol. (2015) 16:101. 10.1186/s12882-015-0090-9 26156092 PMC4496884

[22] Dustin ML Olszowy MW Holdorf AD Li J Bromley S Desai N. A novel adaptor protein orchestrates receptor patterning and cytoskeletal polarity in T-cell contacts. Cell. (1998) 94:667–77. 10.1016/S0092-8674(00)81608-6 9741631

[23] Takano T Bareke E Takeda N Aoudjit L Baldwin C Pisano P. Recessive mutation in CD2AP causes focal segmental glomerulosclerosis in humans and mice. Kidney Int. (2019) 95:57–61. 10.1016/j.kint.2018.08.014 30612599

[24] Tossidou I Teng B Worthmann K Muller-Deile J Jobst-Schwan T Kardinal C. Tyrosine phosphorylation of CD2AP affects stability of the slit diaphragm complex. J Am Soc Nephrol. (2019) 30:1220–37. 10.1681/ASN.2018080860 31235616 PMC6622410

[25] Shih NY Li J Cotran R Mundel P Miner JH Shaw AS. CD2AP localizes to the slit diaphragm and binds to nephrin *via* a novel C-terminal domain. Am J Pathol. (2001) 159:2303–8. 10.1016/S0002-9440(10)63080-5 11733379 PMC1850607

[26] Schwarz K Simons M Reiser J Saleem MA Faul C Kriz W. Podocin, a raft-associated component of the glomerular slit diaphragm, interacts with CD2AP and nephrin. J Clin Invest. (2001) 108:1621–9. 10.1172/JCI200112849 11733557 PMC200981

[27] Shih NY Li J Karpitskii V Nguyen A Dustin ML Kanagawa O. Congenital nephrotic syndrome in mice lacking CD2-associated protein. Science. (1999) 286:312–5. 10.1126/science.286.5438.312 10514378

[28] Kim JM Wu H Green G Winkler CA Kopp JB Miner JH. CD2-associated protein haploinsufficiency is linked to glomerular disease susceptibility. Science. (2003) 300:1298–300. 10.1126/science.1081068 12764198

